# Public Interest Group on Cancer Research: a successful patient–researcher partnership in Newfoundland and Labrador

**DOI:** 10.1186/s40900-022-00380-8

**Published:** 2022-09-03

**Authors:** Sevtap Savas, Holly Etchegary, Teri Stuckless, Cindy Whitten, Jason Wiseman, Derrick Bishop, John King, Janine Cutting, Darrell Peddle

**Affiliations:** 1 Division of Biomedical Sciences, Faculty of Medicine, Memorial University, 300 Prince Philip Drive, Room 5M324, St. John’s, NL Canada; 2 Discipline of Oncology, Faculty of Medicine, Memorial University, St. John’s, NL Canada; 3 Public Interest Group on Cancer Research, St. John’s, NL Canada; 4 Division of Community Health and Humanities, Faculty of Medicine, Memorial University, St. John’s, NL Canada; 5 NLSUPPORT, Faculty of Medicine, Memorial University, St. John’s, NL Canada; 6 Provincial Cancer Care Program, Eastern Health, St. John’s, NL Canada; 7 Research and Innovation, Eastern Health, St. John’s, NL Canada

**Keywords:** Cancer, Caregivers, Diversity, EDI, Family members, Newfoundland and Labrador, Patient partners, Patient partnership, Patient-oriented research, Public engagement

## Abstract

**Background:**

Partnering with patients and family members affected by cancer is essential for meaningful research, public engagement and outreach, and advocacy activities.

**Objective:**

Our objective was to create a public interest group through an academic–community partnership focused on cancer research and public engagement.

**Methods:**

A purposeful recruitment process was implemented to ensure a diverse and inclusive group. The group meets virtually and communicates by email. The group’s activities focus on identifying the needs, priorities, and interests of cancer-affected individuals in the province; consultations; and designing research projects and public outreach activities together. Comprehensive meeting minutes are kept and used to distill discussion points. The work of the group is disseminated through a variety of channels.

**Results:**

The public interest group includes 12 cancer patient and family member representatives, in addition to researchers. Discussions by the interest group identified key themes related to: (1) equity issues and regional disparity in provincial oncocare; (2) information needs; (3) need for patient empowerment and public understanding; and (4) family member and partner needs and experiences. To date, the group has co-designed a cancer research proposal and a public engagement/outreach activity. The group also provides consultations on cancer-related projects/public engagement activities and members act as patient partners in specific research and public engagement proposals. The group evolves over time, and increasingly advocates on behalf of cancer patients and families. Retention and satisfaction of the public partners with group activities have been high. The group’s work and findings are disseminated to the Provincial Cancer Care Program, as well as to public and scientific stakeholders through local media, academic conferences and presentations, and a dedicated website.

**Conclusion:**

Public Interest Group on Cancer Research represents a highly successful patient–researcher partnership in oncology. It designs meaningful and patient-oriented studies and outreach activities in cancer. It also elevates and widely supports cancer patient and family voice.

**Supplementary Information:**

The online version contains supplementary material available at 10.1186/s40900-022-00380-8.

## Plain English summary

Cancer patients and their family members have unique and lived experience with the condition. Therefore, collaborating with them is important in cancer science. We aimed to create such a collaboration in Newfoundland and Labrador, Canada. In 2021, we successfully formed our diverse collaborative group. Currently, our group includes 12 public representatives. We meet online and discuss matters important to members. We also design studies and events together. Our discussions have identified four topics that need further research and policy changes such as information needs and unique needs of caregivers and family members. Our activities expand over time. For example, lately we started to advocate for other cancer patients and families. In conclusion, we formed a successful cancer patient, family member, and researcher collaborative group. Our work informs the public, healthcare systems, and scientists on important cancer related matters.

**Supplementary Information:**

The online version contains supplementary material available at 10.1186/s40900-022-00380-8.

## Background

Recent years have witnessed a growing emphasis on patient-oriented research (POR), patient and public engagement (PE) and outreach [1–3].

Since its inception in 2011, the Canadian Institutes of Health Research (CIHR) Strategy for Patient-Oriented Research (SPOR) has been a driver of health-research related patient engagement and POR practices in Canada, with infrastructure and initiatives developed across provinces and health disciplines [4]. Dedicated to building the capacity for POR and patient/public engagement, SPOR established Provincial SPOR SUPPORT Units, whose role is to champion POR and build POR capacity in local priority research areas. At the centre of SPOR is the involvement of patient/family member/caregiver in health-related research, so that researchers can conduct meaningful studies that will improve patient outcomes and the healthcare they receive [5].

Worldwide, public engagement and outreach are common goals of academic institutions [6, 7]. Public engagement and outreach efforts aim to connect, engage, and work with the public members (and patients and families) towards a variety of goals, such as to disseminate and exchange knowledge, and to integrate public and community members’ opinions in academic, scientific, and community based activities and studies. Depending on the aim of the partnership, public engagement and patient-oriented research activities can go hand-in-hand, for example, in the case of cancer.

There is quite an interest for patient and public partnership in cancer. In this manuscript, we describe the aims, creation, and work of such a partnership—the Public Interest Group on Cancer Research (“PI group”). This group was created in 2021 in the Canadian province of Newfoundland and Labrador (NL). NL is located in the eastern Atlantic region of Canada, and consists of the island of Newfoundland and mainland Labrador. The province has a population slightly above 500,000—with the majority residing on the island [8]. Around 11% of the population identifies as Indigenous, whereas European descendants make the largest ethnic group [9]. NL has an above national average proportion of rural communities (around 40%) [10], which have, in recent decades, experienced economic down-turn [11]. This and the aging population in the province have implications for cancer rates and cancer-related mortality. According to the Canadian Cancer Statistics 2021 report [12], residents of NL have the highest cancer risk and mortality rates in the country. Every year, around 3950 NL residents are diagnosed and around 1590 NL residents die of cancer. The impact of the geographically distributed sparse population on the social determinants of health, including access to education and healthcare services, may have a role in these cancer rates. Provincial healthcare is provided through four regional health authorities (Eastern, Western, Central, and Labrador-Grenfell health authorities). Most of the cancer surgeries and treatment are performed in facilities in Eastern Health located in the capital city of St. John’s. This means a significant portion of the rural patient population must travel for cancer treatment and management, and access to healthcare and support services may be an issue.

The NL population is quite responsive to research. There have been many successful studies conducted in the NL population, which sometimes shed light on the diseases substantially and provided relief to families (e.g., [13–15]). Memorial University (MUN) is the only university in the province. MUN has “a special obligation to the people of NL” [16], and has a very successful history of community-university partnership. In 2012, MUN also implemented a very strong Public Engagement (PE) framework [17], created a dedicated PE office, and offers PE awards and grants to support and encourage public/community–researcher partnerships. In addition, MUN’s Faculty of Medicine hosts the NLSUPPORT unit [18], the CIHR SPOR unit dedicated to support the patient-oriented research activities in the province. At the government level, public engagement is also encouraged and valued in decisions related to provincial healthcare, as exemplified by the Health Accord NL, a provincial task force that was created in 2020 in order to “reimagine” the future of population’s health outcomes and healthcare in the province [19].

The striking rates and impact of cancer on the community, the public’s interest to collaborate with MUN researchers for the benefit of the population, together with the support by MUN’s PE framework and NLSUPPORT have allowed us to create the Public Interest Group on Cancer Research. The overall goal of this group is to make a positive difference in the lives of cancer patients and families in NL through designing and conducting cancer research studies, public engagement-outreach-education activities, and advocacy on behalf of others *together*. While patient and family councils have been created in healthcare organizational initiatives [20–22], to our knowledge, no other patient-academic partnership that focuses on cancer with such a broad and dynamic scope exists elsewhere in the country. In this manuscript, we describe this group’s characteristics, its work, and experiences and lessons learnt along the way by its members.

## Methods

### Founder members

These members initially included Sevtap Savas (SS; a cancer scientist; MUN), Holly Etchegary (HE; a health and social scientist; MUN), Teri Stuckless (TS; an oncologist; MUN; Eastern Health), Farah McCrate (FM; a healthcare administrator; Eastern Health), Rebecca Roome (RR: a community member), and Doug Smith (DS: a community member). In 2022, TS, FM, RR, and DS stepped down due to life changes or new professional roles. Cindy Whitten (CW) then joined the group as the Eastern Health representative and a scientist. Like any other group member, the founder members attend the group meetings, help finalize the meeting minutes and agenda items, and help with public outreach and proposal development. They additionally have the administrative roles (e.g., in grant writing, submissions and management) and work on most of the dissemination activities.

### Recruitment of public partners

Recruitment ads were circulated through the Office of Public Engagement website, social media, personal contacts, local media news/interviews, MUN listserv, and NLSUPPORT newsletter. Residents of NL 18 years or older, diagnosed with cancer or had a family member diagnosed with cancer, and could meet virtually/through phone were invited to complete a short survey collecting their basic demographic, geographic, and disease-related information. This information was needed to make the final selection of the group members with a focus on equity, diversity, and inclusion (EDI).

In ~ 3 weeks, 42 applications were received—at which point the recruitment process was stopped. Information collected from the applicants was utilized to select the final 12 public members to maximize the EDI (i.e., to ensure under-served communities were involved and a range of cancers, ages, sexes, geographic areas and patient/family perspectives were included). The selection process was made independent of the founding members by a research staff member. In addition to 12 public members, the final group also included the founder members. At the end of the 1st year, two members had to step down due to professional commitments or personal reasons. This led to the recruitment of two new public members in 2022 through social media or personal contacts, and the total number of public partners remained the same (n = 12).

### Meetings

All meetings are held virtually through the WebEx platform (vetted by Memorial University), with the option of participating via phone. The first meeting of the interest group was held in May 2021. The Terms of Reference document—adapted from a local patient advisory committee—was reviewed and finalized to guide the group operations. Meetings usually last around 1.5 h with the total number of meetings in 2021 being four. In 2022, meetings were held on the average once a month with a break in summer months, as per the public partners’ request at the end of the first year. Meetings were not recorded; rather comprehensive notes were taken and minutes documents were circulated to the entire group within a week. Agenda items and other necessary materials were sent to the group a week before, and reminder emails were sent on the day of the meetings. When needed, additional communications were made through email, and rarely, through phone correspondences. The meetings were chaired and Meeting Minutes were drafted by SS. The public members had the option to co-chair the meetings. Respect for each group members’ opinion and contributions was continually displayed. Members were reminded at the beginning of each meeting that they did not have to share any information they were not comfortable sharing. Members also have had the option to request individual meetings should they not be comfortable talking within a group setting (e.g., on sexual health matters). Also, some meetings have utilized the option of virtual breakout rooms via WebEx to help facilitate dialogue between group members.

### Group discussions and key points

Each meeting has served to socialize, discuss needs of cancer-affected individuals and family members/consultations, and plan the group’s next steps. In 2021, the group’s discussions focused broadly on the needs and priorities of cancer patients and families in the province. Discussions were guided through “guidance questions” and discussions by the public members were prioritized/encouraged (e.g., discussions by scientists/founders were discouraged). Members were also advised to think about the experiences of the patients and families in their communities and close social circles (i.e., not only their own experiences). At the end of 2021, the discussion points collected and noted in the Meeting Minutes were digested, classified, and presented to the entire group. In 2022, the group’s focus has expanded to caregiver needs, sexual and mental health needs, advocacy, and organization of the group’ own public engagement project—*Public Conference on Cancer*. Upon request by a cancer survivorship working group within the Eastern Health, the group also provided consultations and a brief report on sexual health matters experienced by cancer patients to help organize future webinars/patient education sessions.

### Dissemination plan and activities

The PI group aims to widely disseminate its work and patient/family member perspectives, needs, opinions, and priorities with all stakeholders and community-at-large. In 2021, a report was produced describing the aims and work of the PI group, which was sent to the Health Authority leadership, director of the Provincial Cancer Care Program, and the Chair of Oncology at MUN. Social media as well as local media articles and radio program interviews were utilized to disseminate the group’s work and findings, engage with the members of general public, increase awareness (e.g., about the disease symptoms, importance of screening and early diagnosis, patient and family member lived experiences), and to advocate for cancer patients and families. Interested public partners were included in the media interviews, as well as in drafting media articles [23–27]. We have also presented (or will be presenting) the group’s work in academic settings, including in cancer conferences (Canadian Centre for Applied Research in Cancer Control [ARCC] conference held on May 2022 [28]; International Psycho-Oncology Society/Canadian Association of Psychosocial Oncology [IPOS/CAPO] conference to be held in August–September 2022 [29]), and in the Oncology Grand Rounds—MUN/Eastern Health in June 2022. Last, in 2022, we also started a dedicated webpage to disseminate and discuss the work of the public interest group [30].

### Satisfaction survey

An online survey was implemented in Qualtrics and presented to the public members in the group in December 2021–January 2022. The participation was voluntary and all responses were anonymous. Responses were summarized and discussed by the group. Among the member feedback was a preference for monthly meetings (rather than quarterly) and using the meeting time to also promote engagement and socialization within the group, which were implemented.

### Compensation

In alignment with the CIHR SPOR guidelines [31], we offered a one-time honorarium to each public member of the group in 2021 ($150) and 2022 ($200).

## Results

The work of the Public Interest Group on Cancer Research has led to many important developments, initiatives, and activities in Newfoundland and Labrador.

We have successfully formed a cancer patient/family and researcher/healthcare administrator partnership in NL. This partnership has included individuals from three important organizations and stakeholders—MUN (e.g., researchers), Regional Eastern Health Authority (e.g., research department administrators/researchers), and community members (i.e., cancer patients and their family members). Dialogues among these three are needed for shared understanding, informing research and healthcare, ultimately leading to better outcomes for patients and families affected by cancer [4, 5]. Since all sides share common goals, forming such a bridge has been an important part of the work. Additionally, such a multi-stakeholder effort has been a catalyst for patient and family needs as well as perspectives to be widely recognized.

The purposeful recruitment strategy has helped us form and keep the Public Interest Group on Cancer Research as a highly diverse, inclusive, and equity-oriented group (EDI; Table 1). Utilizing EDI principles is needed, not only because it ensures hearing the perspectives of traditionally under-served communities, but also because cancer experiences vary depending on, for example, sex/gender, cancer site, ethnicity, geographic location, and other factors. The discussions and activities of the group are therefore informed by a diverse, rich representation. While members from urban areas of the province were over-represented, and as observed in other committees [1], men have been slightly under-represented, the group has had a good representation in terms of cancer sites (more than 14 different sites as well as select common and rare cancers being represented); hereditary versus sporadic cancers; cancer patient and family members; ethnicity (i.e., white, Indigenous, Asian); self-identified disability status; and the age groups (between 18 and 65+ years of age). In the 2nd year, we did not have representation from Labrador—efforts to integrate these perspectives in the group are ongoing. However, in 2021 we were also able to recruit a member of the 2SLGBTQIA+ community.

**Table 1:** Equity, diversity, and inclusion characteristics of the Public Interest Group on Cancer Research (2021–2022)

Variable	2021	2022
Sex		
Men	4	5
Women	8	7
2SLGBTQIA+ member	0	1
Location		
Rural	4	4
Urban	8	7
Don’t know	–	1
Newfoundland	11	12
Labrador	1	0
Ethnicity		
White	8	9
Indigenous	3	2
Asian	1	1
Disability		
Members with disability	4	4
Hereditary cancers		
Members with familial/hereditary cancer history	4	4
Age		
Age groups of members	18–66	18–66+
Cancer sites represented		
Cancer sites in the family	14*	18**
Members who have had a cancer diagnosis		
Cancer patient/survivor	5	7
Members with a family member diagnosed with cancer		
Family member of a cancer patient/survivor	10	10

Numbers exclude the founder members of the group. Age groups include: 18–25, 26–35 with 10 years increment, up to 66+

*Includes skin, lung and bronchus, liver, cervical, ovarian, pediatric, blood, kidney, prostate, bladder, colorectal, thyroid, and brain/central nervous system cancers

**Includes all but blood cancers in *, in addition to esophageal, oral/head and neck, pancreas, stomach and uterus cancers

**Fig. 1:**
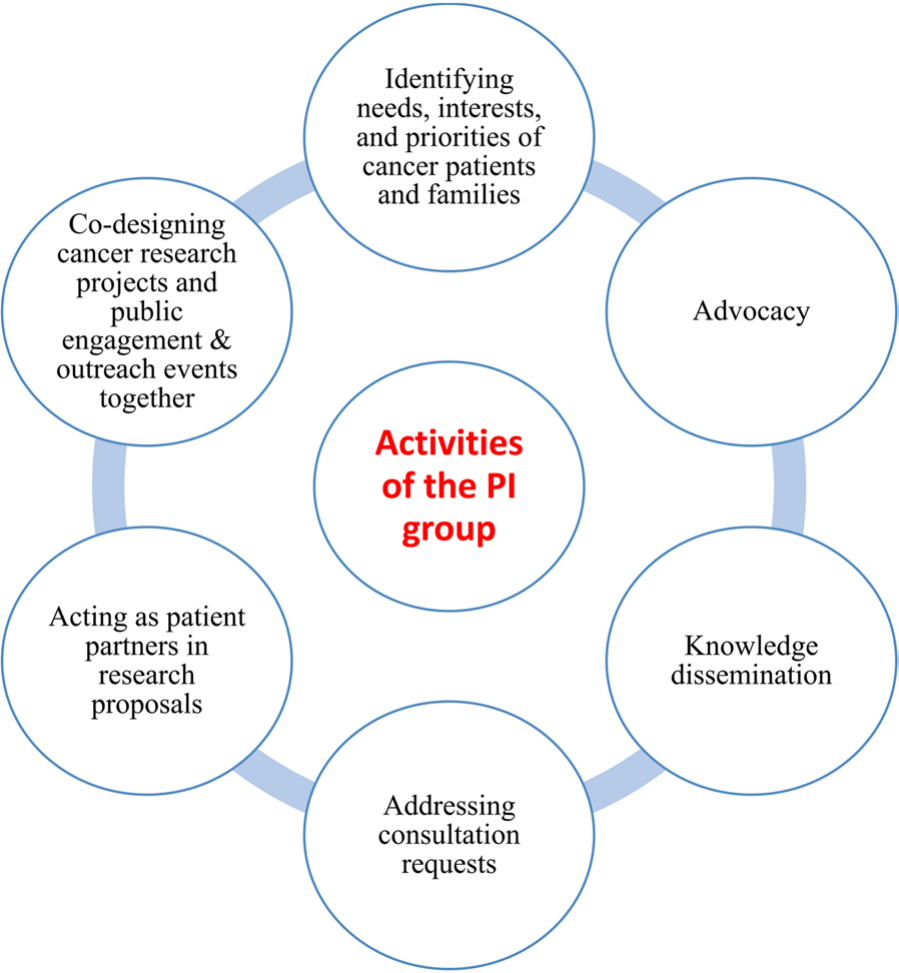
Main types of activities of the Public Interest Group on Cancer Research. *NL* Newfoundland and Labrador, *PI group* Public Interest Group on Cancer Research

In terms of the activities, this interest group has developed, progressed, and initiated several important ideas (Fig. 1). The patient partners act as experts of cancer lived experiences, citizen scientists, patient partners, consultants, speakers, authors, or advocates in these activities.

**Fig. 2:**
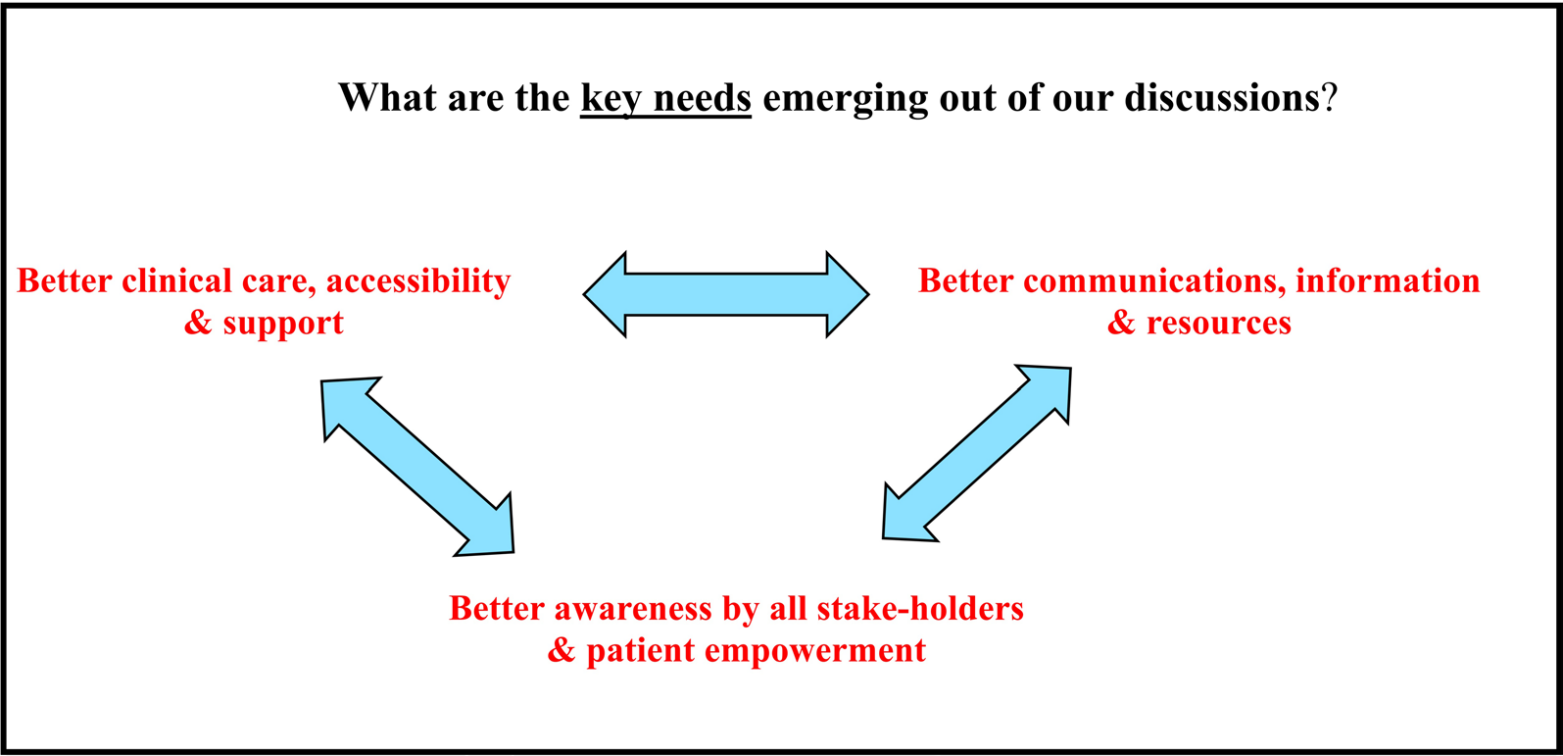
Key knowledge extracted from group discussions in Year 1—themes identified

**Fig. 3:**
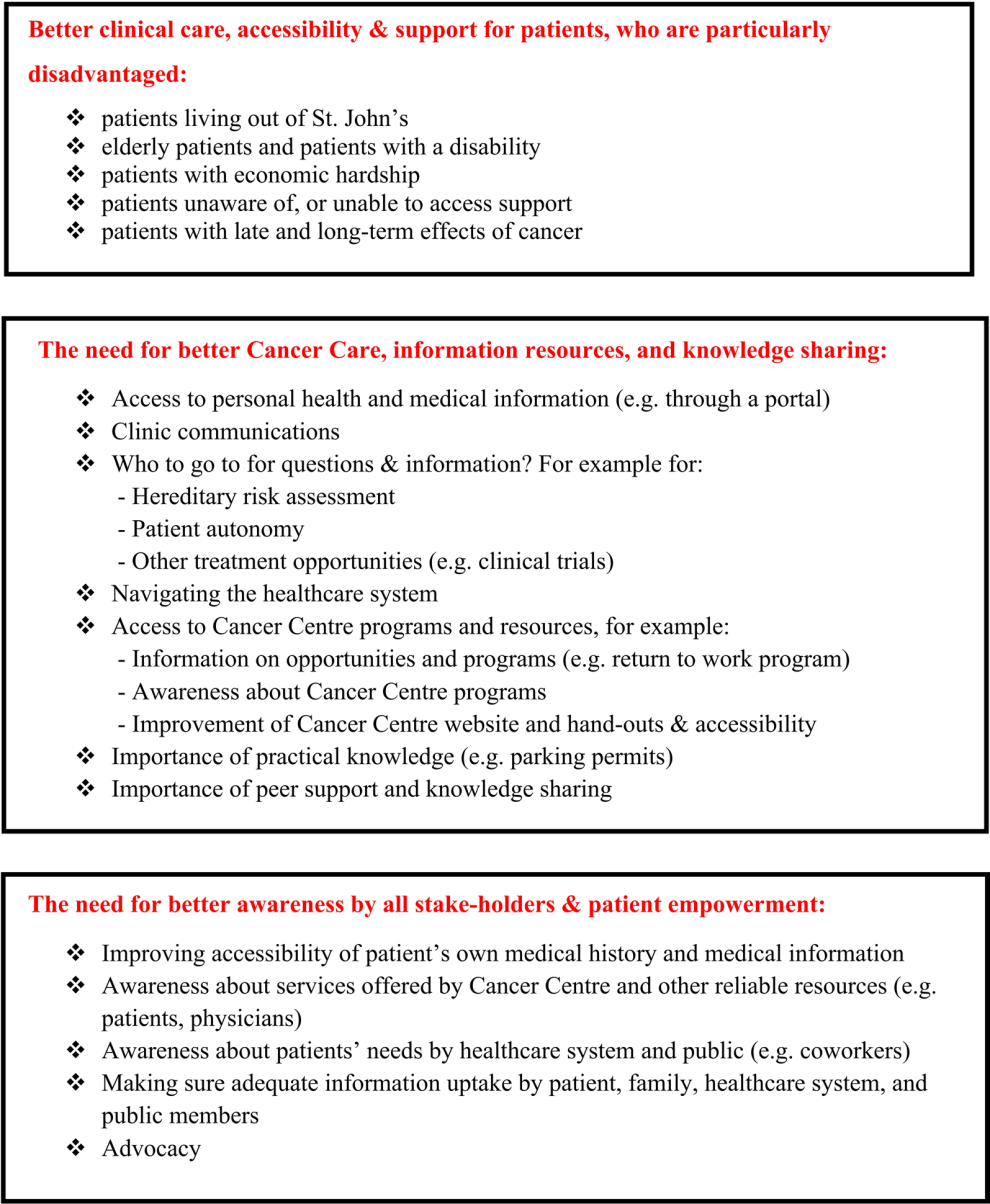
Key knowledge extracted from group discussions in Year 1—examples
  In the second year, the group discussions moved to the caregiver/family member perspectives and sexual health needs (the topic of a requested consultation—see below). These conversations generated important insight towards the “familial” nature of the disease, including the varying types of disclosure preferences by family members, as well as the support needs of caregivers in the family. In terms of sexual health, the main perspective was the inefficient communication regarding sexual health matters in cancer care (including long or late effects of treatment) and interventions that can help alleviate sexual health issues (e.g., hormone therapy in treatment-induced early menopause). Sexual health matters also included the counselling and support needs of spouses/partners. There were additionally concerns around the reluctance of men to talk about their sexual health and body image issues that may be experienced and how to address them during the cancer journey (Fig. 4).

1. Identifying the needs, priorities, and interests of the cancer patients and family members in NL: The first year of the group focused on these important themes and aimed to synthesize the key points from the discussions. As a result, three key topics were identified (Figs. 2, 3). The most common and comprehensive need was around information needs. The group members were also aware of the regional disparity (rural versus urban areas; Labrador versus Newfoundland) and equity issues (for example, age- and disability-related access issues), and wished for equal access to health and cancer care in the province. Last, patients also expressed the need for empowerment—in terms of accessing information or services they may need/benefit from—as well as better understanding by “others” of their life changes, perspectives, and experiences following a cancer diagnosis.

**Fig. 4:**
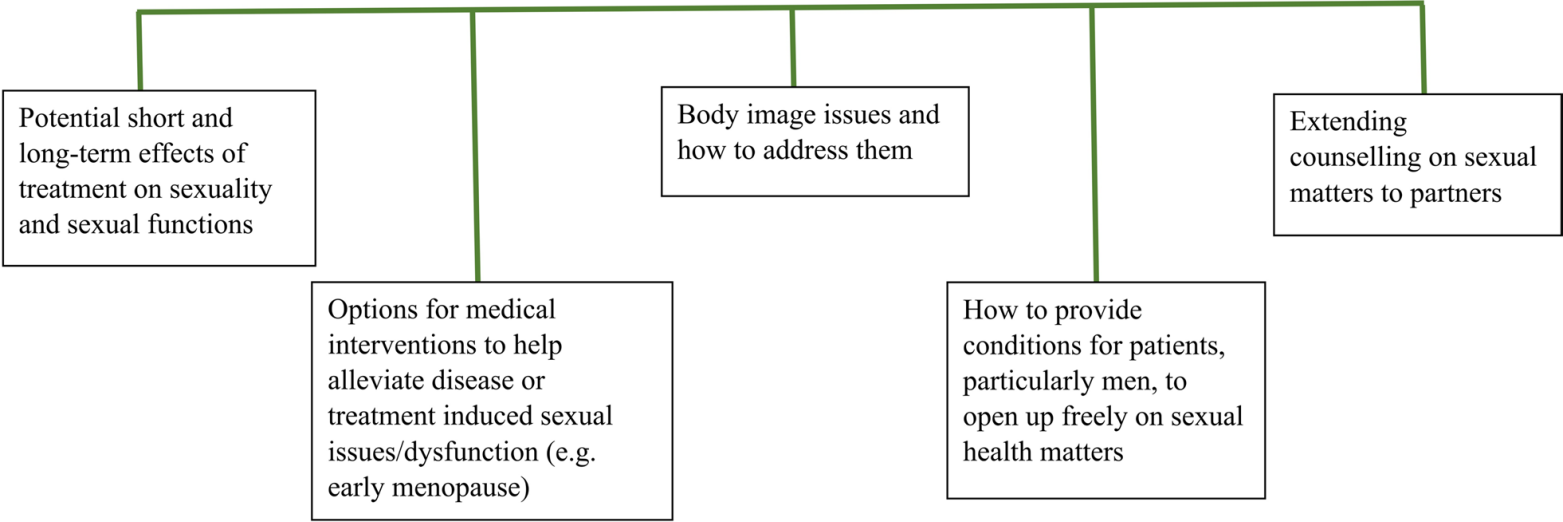
Sexual health matters from the patient and family member points of view
2. A research project solely based on the group’s discussion prioritizing patient and family needs was developed. The entire group—including cancer patients and family members—therefore act as scientists in this project. This project aims to improve the access of reliable information needed by cancer patients and families throughout their cancer journey. It also involves collaborations with the key players in healthcare/cancer care system, and as such, is an important project that can help translate the findings into clinic.
3. The group has developed its own public engagement and outreach activity for Fall 2022—a virtual *Public Conference on Cancer*. This conference idea arose from the group’s discussion about the lack of knowledge or awareness of important information, such as on cancer screening programs offered to the population of NL, specific support services and care programs offered by the Provincial Cancer Care Program (e.g., nurse navigator program; Lost in Transition program), and patient and family members lived experiences and perspectives. The patient and public-informed and patient-oriented nature of this Conference is expected to have a strong role on its successful uptake and attendance.
4. Patient representatives are invited to form teams with researchers as patient partners in local cancer-related projects.
5. The group actively and consistently disseminates their work and opinions, and advocates on behalf of others. Three main channels of dissemination have been utilized so far:
  - *Local media—including articles and radio interviews* In 2022, these activities also included the interested patient representatives in the group. Communicating both the scientist and patient partner perspectives simultaneously provides a unique opportunity. Involvement of the public partners empowers them and the communications: in our experience they connect and convey important messages to the audience/public in a way that may not be possible for scientists (or healthcare administrators and providers).
  - *Academic and healthcare settings* Public engagement and patient/public-oriented research are important parts of scholarly work. As of June 2022, the PI group presented their work in one national cancer conference (ARCC) (another abstract accepted for an international conference; IPOS/CAPO) in 2022, and in an Oncology Grand Round with participants including both oncologists and researchers. Since the participants included healthcare providers, this last presentation was critical to communicate patient perspectives directly with the local cancer care system while also using academic lenses. Additionally, we voluntarily submit an annual report to leadership at Regional Eastern Health Authority as well as the Provincial Cancer Care Program, with the hope that the group’s discussions, findings, and solutions offered will be useful for their mandates and operations.
  - *Social media and website* Some of the members of the public interest group disseminate knowledge through their social media accounts for the larger community. The group also posts and disseminates knowledge through a dedicated website [30].
6. As the work of the group was being disseminated, we began to receive consultation requests. For example, the group was asked to identify the sexual health and wellness needs of cancer patients and significant others. This example demonstrates that the group is recognized as an effective patient and family voice in the province. It is important to note that this information was utilized by a survivorship working group established within the healthcare system. Therefore, it is a particularly positive development in terms of the link between this group and healthcare providers/administrators.
7. Advocacy activities. The interest group is increasingly involved in advocacy on behalf of others who cannot voice their concerns, experiences, or perspectives. These advocacy activities include not only social and mainstream media appearances and articles, but also communicating patient needs to provincial government and elected officials, and calls for more action [24–27].

## Discussion

Here we describe the aims and work of Public Interest Group on Cancer Research, a highly successful cancer patient–family member–researcher partnership in oncology. The way this public interest group is created and operates may encourage others to start similar successful partnership in cancer and other diseases.

The main goal of this group is to improve the wellness and conditions of cancer patients and families through designing and conducting new research, public engagement & outreach activities with the spirit of genuine partnership. Group members are dedicated, enthusiastic, and interested in contributing to its goals. The second year, particularly, has witnessed clarity around our work/aims, interest and involvement of public members in advocacy, media interviews, and articles, and getting stronger in discussing the matters of highest priority to the community. The group evolves in its activities and discussion topics. Over time, the group has also adopted advocacy as one of their activities. Helping others going through cancer and changing conditions (e.g. healthcare) for the better were the main motivations for public members to join this group. For example, one member’s response to the question of why they joined this group was to “*Share my perspective on the cancer experience in NL and hopefully help shape the way others' experiences will be in the future*”. As another member said "*I expect that my own experience coupled with a positive approach to problem-solving can contribute to the goals of the group. My experience so far is that the group is highly inclusive, well-motivated and expertly led. These attributes are already yielding dividends in recognition and support by other organizations provincially*.”. These also suggest that in at least some cases, the patient partners appreciate that their lived experience is integral to change and improve the conditions of cancer-affected individuals (Additional file 1).

The interest group is unique in the sense that it integrates public engagement, patient-oriented research, and advocacy at the same time. Its work and scope are dynamic, flexible, and evolve. Importantly, the group membership has been established and maintained with an EDI focus; therefore, it is as inclusive as possible and contains diverse cancer patients/family members from across the province. This EDI focus helps us to hear and work with particularly the individuals from under-served, traditionally vulnerable or equity-deserving communities. The group members also include individuals with different expertise (e.g., cancer scientist; health scientist; healthcare administrator; patient and family member) and lived experience of cancer (e.g., self or family member). All of these enable us to complement each other’s perspectives, exchange valuable and diverse knowledge, and generate new ideas. This feature of the group has greatly helped us distill knowledge and develop ways to address key priorities through scholarly activities, for example by co-designing research studies and public events together. Overall, the group is diverse, enthusiastic, responsive, and open to act on discussion points and public member recommendations. As one member expressed during a radio interview “*when we come together, we always find new things to do—to make things better for the next people*”. This sentence summarizes the spirit of this partnership.

The dynamic and rich group conversations have helped us to identify a number of topics to work on. Our group utilizes this knowledge to design studies and public events. We also very liberally disseminate this knowledge, including in scientific venues, social and local media, as well as, as presentations and reports voluntarily submitted to relevant healthcare units and leadership in Newfoundland and Labrador. Therefore, this group utilizes various ways to inform the academic units, healthcare systems, and the general public in order to facilitate understanding of their perspectives and lived experiences, and to motivate further action and improvement in the lives and outcomes of cancer-affected individuals. We note that the recent provincial Health Accord NL recommendations too emphasize the importance of inclusion, patient and community-centered healthcare, improving social determinants of health, and realigning the services based on patient and population needs [19]. We are confident that over time the PI group will continue to play important roles to bring patient and family voices from across the province, advocate on behalf of all, and help implement positive changes in alignment with the Health Accord recommendations.

Looking forward, we would like to adapt to changing conditions and continue to address group’s priorities. Public Interest Group on Cancer Research has been created during the COVID-19 pandemic. Therefore, the entire group meets and functions virtually and through emails. This may mean that we are missing representation by individuals who do not have access to phone, email, internet, or computer (or do not have computer skills. Low internet bandwidth as well as poor cell phone coverage/services are known problems in certain parts of the province [32, 33]). On the other hand, the virtual meeting format also allowed us to include members from across the province. This could not be possible by in-person meetings. Considering these two points together, moving forward, we are planning to implement a hybrid format for our meetings when it is safe. This option was suggested by the public partners in the group. We also would like to continue to reach out and recruit representations that we are currently missing, including individuals from Labrador. Additionally, we would like to continue to strengthen and expand our collaborations with the healthcare system, particularly the Provincial Cancer Care Program, and collaborate more extensively. This is an important task and aim for our group.

Last but not least, the group discussions were very helpful in terms of identifying medical, social, geographic, and information-related barriers and issues to be addressed in NL. These include the geographic disparity and inequity in accessing medical care and Cancer Care Program’s services; improvements needed to serve the vulnerable individuals; counselling and support needs of partners/caregivers; the need for better sexual health support; and the need for efficiently informing the patients and families—not only regarding disease, treatment, prognosis, or end-of-life related topics, but also regarding support services available to them. Conducting studies on these barriers and limitations, and implementing communication and additional relevant plans in the healthcare system are expected to help improve the health outcomes of cancer-affected families, and therefore, should be encouraged.

### Lessons learnt and opportunities

There are several opportunities identified and lessons learnt during this partnership.

From the founders’ point of view, responses to the first satisfaction survey were very helpful to understand patient partner experiences and preferences in terms of how to operate as a group. We will continue with an anonymous survey each year, and are reminding the partners to freely bring forward and discuss any issues or opportunities they see during the group meetings.

The annual honorarium was generally welcomed, but could also give the impression of “expectations” from the public members. Hence, emphasizing what honorarium is (e.g., a small token of appreciation, with no expectation attached to it)—early and often—can help prevent such a burden placing on public partners.

Finding a time to meet for a group as large as this interest group (15–18 members) can be challenging, but has been going well with attendance rates between 50 and 89% (minimum 9 attendees). However, it became apparent early on that certain months of the year were not good times for meeting, such as December—early January as well as summer months, considering these are the times that people often spend with their family, or go on vacation. Therefore, if there are time-sensitive matters to discuss, optimum timing of the meetings needs to be considered.

The enthusiasm and genuine interest of the cancer patients and families helping other cancer families is very strong. Many of them are also very comfortable in sharing their perspectives and communicating with the public members and scientists alike. They represent public members as well, and as such, can connect with the members of the general public at levels that may not be possible by healthcare providers, administrators, or politicians. Messages conveyed by them, such as the importance of symptom recognition leading to early diagnosis; importance of early detection and screening programs leading to better treatment options/effectiveness and increased survival chances; and expression of lived experiences and support/solutions/opportunities experienced during the cancer journey can be an effective part of the public and patient education, and outreach activities.

From the patient and family member point of view, responses to the year 1 satisfaction survey indicated that the group was generally satisfied/highly satisfied with how the group functions and how members are treated, but they also wanted to meet more frequently so that they can get to know each other better. This has been very welcomed feedback and showed that forming a sense of belonging and being a part of a group may be challenging in virtual settings, but can be addressed by increased frequency of meetings and interactions during the meetings (i.e., not just focusing on the work—meeting topics only). They also expressed interest in knowing how they were valued and being asked individually to provide their opinions during the meetings. By adopting a welcoming and respectful environment, group members relay that they feel comfortable in sharing information relevant to the ways in which cancer has impacted them and their families. The public partner feedback is appreciated, taken seriously, and helped the group change how it functions for the better. The team is committed to ensure that the public member comments and feedback are utilized and addressed effectively.

## Conclusions

In conclusion, partnering with cancer patients and families can be a meaningful, exciting, efficient, informative, and dynamic experience. Our work together helps increase the visibility of patient and family member perspectives; inform the academic units/researchers, healthcare systems/providers, and general public; and design meaningful cancer research studies and public engagement activities that will address the most pressing needs and priorities identified by patients, in line with the CIHR’s Strategy for Patient-Oriented Research [4]. Our work also shows that patient empowerment and increased awareness about the needs of cancer patients & families by all stakeholders are needed in NL. Through continuation of our work, and by widely disseminating our work, collaborations, and advocacy, we believe that our group has the ability to contribute to these and other potential positive changes in the province.

## Supplementary Information

**Additional file 1.** GRIPP2 short form. GRIPP2 reporting checklist.

## Data Availability

All data is presented in the manuscript.

## Abbreviations

CIHR: Canadian Institutes of Health Research
EDI: Equity-Diversity-Inclusion
MUN: Memorial University
NL: Newfoundland and Labrador
PE: Public Engagement
PI group: Public Interest Group on Cancer Research
POR: Patient-Oriented Research
SPOR: Strategy for Patient-Oriented Research
