# Supplementary material for: Public Interest Group on Cancer Research: a successful patient–researcher partnership in Newfoundland and Labrador
Source: Res Involv Engagem. 2022 Sep 3;8:46. doi: 10.1186/s40900-022-00380-8 (PMC9440646; doi:10.1186/s40900-022-00380-8)
Supplement: Supplementary file 1 — Additional file 1. GRIPP2 short form. GRIPP2 reporting checklist. [file 40900_2022_380_MOESM1_ESM.pdf]

Additional File 1. GRIPP2 short form

From: GRIPP2 reporting checklists: tools to improve reporting of patient and public involvement in research

Public Interest Group on Cancer Research:

A successful patient – researcher partnership in Newfoundland and Labrador

Sevtap Savas, Holly Etchegary, Teri Stuckless, Cindy Whitten, Jason Wiseman, Derrick Bishop, John King, Janine Cutting, Darrell Peddle.

Section and topic	Item	Reported on page No
1: Aim	Report the aim of PPI in the study	Page 5
2: Methods	Provide a clear description of the methods used for PPI in the study	Pages 6-7
3: Study results	Outcomes—Report the results of PPI in the study, including both positive and negative outcomes	Pages 7-10
4: Discussion and conclusions	Outcomes—Comment on the extent to which PPI influenced the study overall. Describe positive and negative effects	Pages 10-13
5: Reflections/critical perspective	Comment critically on the study, reflecting on the things that went well and those that did not, so others can learn from this experience	Pages 11-13

PPI patient and public involvement
